# Cloning and Identification of Common Carp (*Cyprinus carpio*) *PI3KC3* and Its Expression in Response to CyHV-3 Infection

**DOI:** 10.3390/cimb46100696

**Published:** 2024-10-21

**Authors:** Xiaona Jiang, Lijing Tian, Wanying Ren, Chitao Li, Xuesong Hu, Yanlong Ge, Lei Cheng, Xiaodan Shi, Zhiying Jia

**Affiliations:** 1 Heilongjiang River Fisheries Research Institute, Chinese Academy of Fishery Sciences, Harbin 150076, China; jiangxiaona@hrfri.ac.cn (X.J.); tianlijing@hrfri.ac.cn (L.T.); renwanying@hrfri.ac.cn (W.R.); lichitao@hrfri.ac.cn (C.L.); huxuesong@hrfri.ac.cn (X.H.); geyanlong@hrfri.ac.cn (Y.G.); chenglei@hrfri.ac.cn (L.C.); shixiaodan@hrfri.ac.cn (X.S.); 2 Key Laboratory of Freshwater Aquatic Biotechnology and Breeding, Ministry of Agriculture and Rural Affairs, Harbin 150076, China

**Keywords:** *CcPI3KC3*, common carp, cloning, structural features, CyHV-3

## Abstract

Phosphoinositide 3-kinases (PI3Ks) are a class of key regulatory factors in eukaryotes that can inhibit viral replication by influencing autophagy. Currently, cyprinid herpesvirus 3 (CyHV-3) poses a serious threat to common carp culture. However, PI3K has not yet been identified in common carp. In this study, full-length *PI3KC3* from common carp (*CcPI3KC3*), consisting of an open reading frame (ORF) of 2664 bp encoding a polypeptide of 887 amino acids, with a predicted molecular mass of 101.19 kDa and a theoretical isoelectric point (pI) of 5.97, was cloned. The amino acid and nucleotide sequences of CcPI3KC3 displayed high similarity to yellow catfish’s (*Tachysurus fulvidraco*) PI3KC3. The tissue expression profile revealed that the mRNA levels of *CcPI3KC3* in the liver, spleen, and head kidney were significantly greater than those in the brain, heart, intestines, gills, eyes, testes, and ovaries of common carp. We compared the expression patterns of *CcPI3KC3* between “Longke-11” mirror carp (CyHV-3-resistant carp) and German mirror carp (non-resistant to CyHV-3) at different times (0, 48, 96, 144 h, 192, 240, 288 h post-infection (hpi)) after CyHV-3 infection. The results revealed that *CcPI3KC3* mRNA expression significantly increased in the early infection stage. In the CyHV-3-resistant mirror carp variety, the relative expression of *CcPI3KC3* was significantly greater at 48, 96, and 144 hpi compared with the nonbreeding strain groups after infection (*p* < 0.001). These results indicate that the full-length *CcPI3KC3* sequence was successfully cloned from common carp for the first time, and it might play an important role in the immune system of common carp against CyHV-3 infection. This study provides a theoretical basis for the molecular mechanism of CyHV-3 resistance.

## 1. Introduction

PI3Ks are a family of lipid kinases that are key molecules in the signal transition pathway through a combination of extracellular signals and cell surface receptors and have the activities of lingine/sourein kinase and phospholidosol kinase. The PI3K family includes eight catalytic subtypes. These catalytic sub-types are classified into three classes on the basis of sequence homology and in vitro substrates. Among them, the most widely studied are class I PI3Ks. All class I PI3Ks are formed by heterodimeric protein complexes with catalytic p110 and regulatory subunits. These different p110 subunits are composed of three IA subtypes (p110α, p110β, and p110δ) and one IB subtype (p110γ). p110α and p110β are generally expressed in the liver, heart, intestines, and mammary glands, but p110δ and p110γ are expressed mainly in immune cells and a few specific tissues, such as neurons and the heart [1,2,3]. The physiological function of class II PI3Ks has not been fully elucidated, and the three II PI3K subtypes (PI3K-C2α, PI3K-C2β, and PI3K-C2γ) have distinct and nonoverlapping cellular effects. However, recent studies have revealed the importance of class II PI3Ks in cell proliferation, survival, and migration [4]. Class III PI3Ks, which have only one Vps34 member and are expressed in every eukaryote, were originally found in *Saccharomyces cerevisiae* mutants with vesicle-sorting defects [5,6]. Vps34 (also known as PI3KC3) can bind to specific proteins to form a variety of functional complexes [7,8], including Beclin1 and autophagy-related 14-like protein (Atg14L) [9,10]. Moreover, the PI3KC3 complex plays an important role in the fusion of autophagosomes and in the formation of the autophagosome bilayer membrane in the early stage of autophagy [11,12].

Common carp (*Cyprinus carpio*) is the third most widely farmed freshwater fish in the world and has an extremely high economic value. The current global annual production of common carp is 4.19 million tons [13]. The culture quantity of mirror carp has increased year by year in the proportion of common carp production. Unfortunately, CyHV-3 poses a serious threat to common carp culture. CyHV-3 can cause up to 100% mortality among common carp populations [14] and has caused considerable economic losses in aquatic products worldwide. Previous studies have shown that the up-regulation of *PI3K* gene expression can reduce host autophagy levels and inhibit infectious spleen and kidney necrosis virus (ISKNV) replication in Chinese perch brain cells [15]. PI3K mediates the activation of interferon regulatory factors to inhibit viral replication [16,17]. So far, the entire genome sequence of common carp has been completed, but the sequence and function of *PI3K* in common carp have not been determined yet. A previous study revealed significant differences in the expression of *CcPI3K* in the transcriptome and proteome data between CyHV-3-infected and healthy groups of common carp [18]. In addition, RNA sequencing (RNA-Seq) has been used to identify transcriptional differences between susceptible and resistant fish in response to CyHV-3 infection [19].

In this study, we first cloned and analyzed the full-length sequence of *CcPI3KC3* and determined the relative expression levels of *CcPI3KC3* in the liver, spleen, head kidney, brain, heart, intestines, gills, eye, testes, and ovaries of common carp. We subsequently compared the expression patterns of *CcPI3KC3* between “Longke-11” mirror carp (CyHV-3-resistant carp) and German mirror carp (non-resistant to CyHV-3) at different times after CyHV-3 infection. These results could help further elucidate the function of *CcPI3KC3* and are highly important for exploring the molecular mechanism of CyHV-3 resistance in common carp.

## 2. Materials and Methods

### 2.1. Experimental Animals

The “Longke-11” mirror carp, a new mirror carp variety resistant to CyHV-3 (registration No. GS-01–001-2022, Ministry of Agriculture and Rural Affairs of the People’s Republic of China), and the German mirror carp (non-resistant to CyHV-3) used in this study were obtained from the Kuan Dian Fisheries Experimental Station of the Heilongjiang River Fisheries Research Institute, Chinese Academy of Fishery Sciences. The “Longke-11” mirror carp was bred from the German mirror carp over four successive generations via both mass selection and molecular marker-assisted selection techniques. All experiments were conducted and approved in accordance with the Animal Care and Use Committee of the Chinese Academy of Fishery Sciences (ACUC-CAFS).

### 2.2. Virus Challenge Experiment

Healthy one-year-old fish (72.13 ± 10.30 g) were randomly selected for the experiment, which involved challenge infection by CyHV-3. CyHV-3 content was measured in randomly selected experimental fish, and CyHV-3 was not detected in any of the tested fish. The challenge experiment started after 2 weeks of incubation. A homogenate solution was prepared from the organs of 10 dying fish (these came from Kuan Dian Fisheries Experimental Station of the Heilongjiang River Fisheries Research Institute, Chinese Academy of Fishery Sciences) that were checked for CyHV-3 infection via real-time polymerase chain reaction (PCR) of the virus genes *TK* and *Sph* following the methods described in the industry standard SC/T 7212.1–2011 [20]. This study followed previously published methods by inducing CyHV-3 infection by adding this homogenate, made from diseased fish guts, to the water in the tanks housing the experimental fish [21]. The CyHV-3 contained in the internal organs from the sick fish was homogenized to generate 100 mL of homogenate. The homogenate, containing 1.5 × 10^7^ copies of CyHV-3 per mg, was titrated by real-time RCR with a standard plasmid containing viral genes. The infected fish tissues were detected by PCR, checking for the virus gene *Sph* and *TK*. Experimental fish were then infected with the homogenate, using 17 mL of homogenate per tank. For the “Longke 11” mirror carp and German mirror carp, the CyHV-3-infected group (given diseased fish tissue homogenate) was paired with the uninfected group (given physiological saline). For each treatment group, three replicates were performed. Each tank (1.6 m × 1.2 m × 0.6 m) contained approximately 1 m^3^ of water, with experimental carp (*n* = 100 fish), and the water temperature was maintained at 25 ± 1 °C. The experimental fish were fed 3% of their body weight daily. After the start of the challenge experiment, infection was continued for 288 h post-infection (hpi) until the fish ceased to die. Mortality was monitored at 288 hpi. The mortality rate of the “Longke No. 11” mirror carp was still significantly lower than that of the German mirror carp [18]. During viral infection, the highest mortality rate was 11%, and the highest mortality rate was 69%. The survival rate of the negative control group was 100%. Head kidney tissue samples (*n* = 10) were collected from the CyHV-3 experimental group and the negative control group “Longke-11” mirror carp and German mirror Carp 7 times at 0, 48, 96, 144, 192, 240, and 288 hpi respectively for subsequent experiments. In addition, liver, heart, brain, eye, spleen, intestine, gill, head kidney, testis, and ovary tissues were collected from healthy German mirror carp (uninfected with CyHV-3, *n* = 3). Fish anesthetic (MS-222, 100 mg/L; Beijing Green Hengxing Biological Technology Co., Beijing, China) was used to anesthetize experimental fish. All collected tissue samples were immediately stored in RNAlater (Thermo Fisher Scientific, Waltham, MA, USA) at 4 °C for 12 h and then transferred to a −80 °C refrigerator.

### 2.3. RNA Extraction and cDNA Synthesis

Total RNA was extracted with an RNeasy Mini Kit (Qiagen, Hilden, Germany), and the concentration of the RNA was detected with an ultramicro spectrophotometer. The OD260:280 ratios of all the RNA samples were between 1.8 and 2.0. The integrity of the RNA was detected by 1% agarose gel electrophoresis. Then, the RNA was reverse-transcribed into complementary DNA (cDNA) according to the manufacturer’s instructions and stored in a freezer at −80 °C. According to the manufacturer’s instructions, using the PrimeScript™ RT kit (TaKaRa, Beijing, China) to synthesize each CDNA sample from 1 µg total RNA.

### 2.4. Cloning and Sequencing of the CcPI3KC3 Gene

Three primer pairs (*CcPI3KC3*–1,2,3) were designed with different conservative PI3K sequences from other fish (KU976461.1, MH795166.1, and NM213306.1). Subsequently, the *CCPI3KC3* sequence was amplified by a rapid amplification of cDNA ends (RACE) kit (Thermo Fisher Scientific, Waltham, MA, USA). A series of reactions were performed, such as first-strand cDNA synthesis from the total RNA of the head kidney tissue of the German mirror carp (uninfected with CyHV-3), amplification of a target cDNA, transformation of junction products, and nested amplification by using the RACE Kit, according to the manufacturer’s protocol. Gene-specific primers (RC947-F3, RC947-F4, RC947-R3, RC947-R4, RC947-RT1, RC947-RT2) were used to amplify the part of the *CcPI3KC3* cDNA from common carp. The joint primers (5.3′ outer, 5.3′ outer, 5.3′ inner, 3′ adaptor and 5′ adaptor) and amplification primer pairs (RC947-F3, RC947-F4) were used for nested PCR and amplification of intermediate sequences, respectively. All amplified PCR products were purified to all amplified PCR products through gel extract, and the obtained DNA fragments are connected to the PMD18-T easy vector (TaKaRa, Beijing, China) and converted into DH5α cells (TaKaRa, Beijing, China). All amplified PCR products were gel-purified via a gel extraction kit (Omega, Norcross, Georgia, USA), and the obtained DNA fragments were ligated into the pMD18-T easy vector (TaKaRa, Beijing, China) and transformed into DH5α cells (TaKaRa, Beijing, China). After incubation of bacterial cells at 37 °C for 12 h, the positive cloning containing the expected size plug-in was sent to Shenggong Ltd. (Sangon Biotech, Shanghai, China) for sequencing. All primers are displayed in Table 1.

### 2.5. Bioinformatics Analysis

On the basis of the full-length sequence of the CcPI3KC3 gene, the amino acid sequence and molecular characteristics of CcPI3KC3 were predicted using DNAStar 7.0 software. The secondary structure of CcPI3KC3 was predicted using Phyre2 and DNAstar 7.0 software. ExPASy (https://web.expasy.org/compute_pi/, accessed on 19 October 2024) was used to predict the molecular weight and isoelectric point (pI) of the amino acid sequence. Then the conservative domain of CCPI3KC3 protein was predicted using the NCBI website (https://www.ncbi.nlm.nih.gov/Structure/cdd/wrpsb.cgi, accessed on 5 August 2024). The signal peptide of CcPI3KC3 was obtained from the SingalP 4.1 website (http://www.cbs.dtu.dk/services/SignalP, accessed on 5 August 2024). The TMHMM server 2.0 (http://www.cbs.dtu.dk/services/TMHMM/, accessed on 5 August 2024) was used to forecast the transmembrane region according to the amino acid sequence of CcPI3KC3. The N- and O-glycosylation sites of the CcPI3KC3 amino acid sequence were identified with the online analysis software NetNGlyc1.0 (http://www.cbs.dtu.dk./services/NetNGlyc/, accessed on 5 August 2024) and Yin Oyang1.2 (http://www.cbs.dtu.dk/services/YinOYang/, accessed on 5 August 2024), respectively. Netphos3.1 (https://services.healthtech.dtu.dk/services/NetPhos-3.1/, accessed on 5 August 2024) was used to predict the phosphorylation sites of the CcPI3KC3 protein. The hydrophobicity and hydrophilicity of CcPI3KC3 were predicted using ProtScale (https://web.ExPASy.org/protscale/, accessed on 5 August 2024). The tertiary structure of CcPI3KC3 was predicted by SWISS-MODEL (https://swissmodel.ExPASy.org/interactive, accessed on 5 August 2024). The amino acid sequence of CcPI3KC3 was compared with those of other species using DNAMAN, and before analysis, the sequence was aligned using Clustalx 1.83. These species included the common carp (*Cyprinus carpio*, C. carpio, PP934634), the yellow catfish (*Tachysurus fulvidraco*, *T*. *fulvidraco*, AND78473.1), the scallop Chlamys farreri (*Azumapecten farreri*, *A*. *farreri*, QFR39795.1), the zebrafish (*Danio rerio*, *D. rerio*, NP_957493.1), the blood clam (*Tegillarca granosa*, *T*. *granosa*, QED42021.1), the ridgetail white prawn (*Palaemon carinicauda*, *P*. *carinicauda*, ALY05366.1), and the tropical clawed frog (*Xenopus tropicalis*, *X. tropicalis*, NP_001119972.1). To determine the molecular evolutionary relationship, an evolutionary tree was constructed on the basis of the *PI3K* mRNA sequences of multiple bony fish, humans and mice. Finally, the MEGA7 program was used to generate the evolutionary tree of *CcPI3KC3*, using the neighbor-joining method with 1000 bootstrap replications [22].

### 2.6. Real-Time Polymerase Chain Reaction (RT—PCR)

TB Green™ Premix ExTaq™ II (Takara, China) was used to detect the expression level of *CcPI3KC3* in the heart, liver, spleen, head kidney, brain, eye, intestine, gill, testis, and ovary tissues from German mirror carp (uninfected CyHV-3). TB Green™ Premix ExTaq™ II was subsequently used to detect the relative expression of *CcPI3KC3* in the head kidney of “Longke-11” mirror carp (CyHV-3-resistant carp) and German mirror carp (non-resistant to CyHV-3) at different times (0, 48, 96, 144 h, 192, 240, 288 hpi) after CyHV-3 infection. Specific primers (*qCcPI3KC3*, Table 1) were designed for RT-PCR based on the full-length cDNA of *CcPI3KC3* in common carp using Primer Premier 5.0. According to the TB Green™ Premix ExTaq™ II instructions, RT-PCR was performed using the ABI7500 system (Life Technologies, Carlsbad). *Beta-actin* (*β-actin*) was used as an endogenous reference gene. *β-actin* has been reported as the most suitable reference gene in mirror carp, and its expression remained highly stable across the samples. Each tissue and group had at least three biological replicates. Each sample repeats three technologies. The relative mRNA expression of *CcPI3KC3* was calculated via the 2^−ΔΔCt^ method [23].

### 2.7. Data Analysis

All the data are presented as the means ± standard deviations (SDs) of at least three replicates. The differences in the trial parameters among fish fed different test diets were analyzed with one-way ANOVA or two-way ANOVA. If significant differences were detected, the multiple comparisons using Duncan’s test inspection were performed. IBM SPSS (version 22.0, IBM Corp., Armonk, NY, USA) was used to analyze the experimental data. All of the data were checked for normal distribution using a one-sample Kolmogorov–Smirnov test and homogeneity of variances using Levene’s test. All experiments were performed at least three times. The statistically significant differences were defined at *p* < 0.05 (* *p* < 0.05, ** *p* < 0.01, *** *p* < 0.001).

## 3. Results

### 3.1. Sequence and Characteristic Analyses

**Figure 1:**
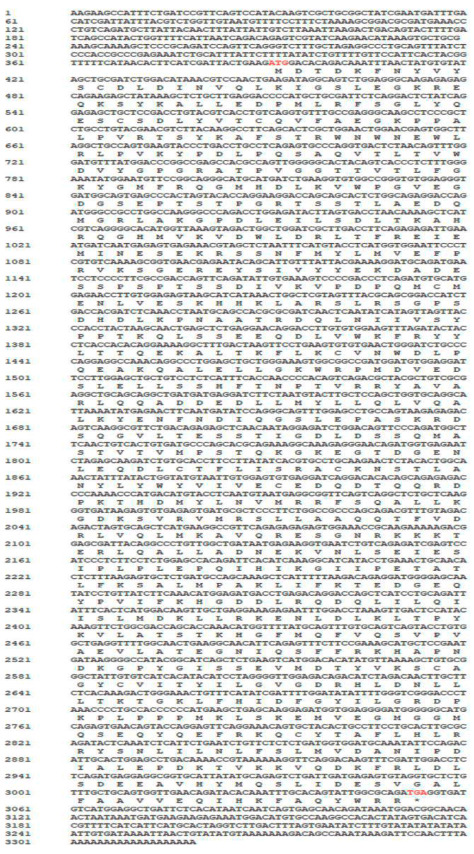
Full-length cDNA and deduced amino acid sequences of CcPI3KC3 in common carp, including the start codon (ATG) and stop codon (TGA). * stands for untranslated amino acids.

The sequencing results revealed that the *CcPI3KC3* full-length cDNA (GenBank number: PP934634), at 3319 bp long, was composed of a 5′-UTR of 390 bp, a 3′-UTR of 265 bp, and an open reading frame of 2664 bp (Figure 1). The ORF was predicted to encode 887 amino acids, the molecular mass was about 101.19 kDa, and an isoelectric point was 5.97. The CcPI3KC3 protein contained 109 strongly basic amino acids (K, R), 120 strongly acidic amino acids (D, E), 283 hydrophobic amino acids (A, I, L, F, W, V), and 238 polar amino acids (N, C, Q, S, T, Y). The instability index of the amino acid sequence of CcPI3KC3 was 46.38.

**Figure 2:**
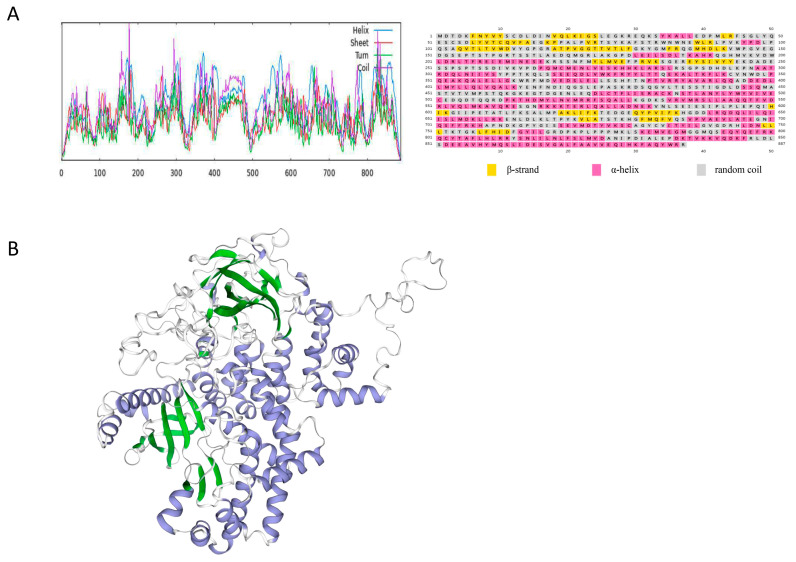
(**A**) Secondary structural prediction of CcPI3KC3 in common carp (α-helix, β-sheet, β-turn, β-strand, random coil). (**B**) Tertiary structure prediction of CcPI3KC3 in common carp (blue represents α-helix structures, green represents β-sheet structures, and grey represents loop structures). The GMQE value is 0.84.

**Figure 3:**
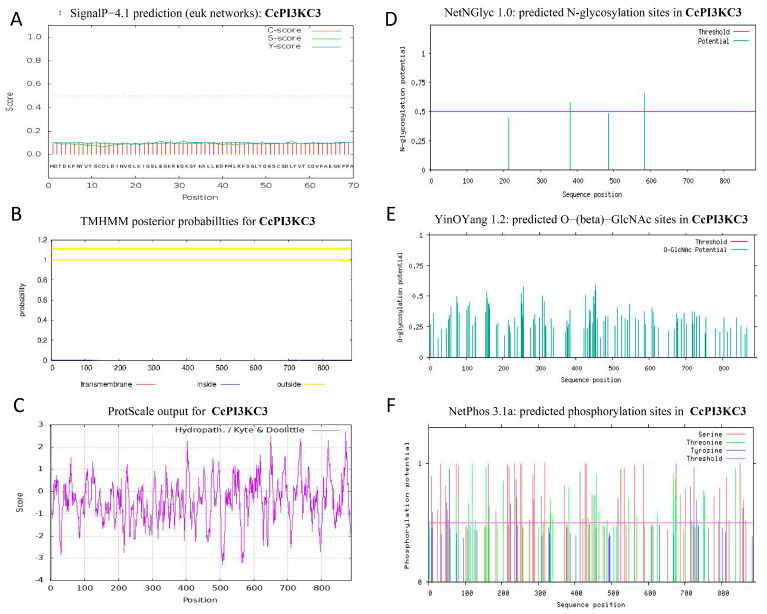
(**A**) Prediction of the signal peptide in the *CcPI3KC3* gene of common carp. The predicted value is marked as the S score. (**B**) Transmembrane domain analysis of CcITGβ1 in common carp (transmembrane, penetrating the cell membrane; inside, intracellular membrane; and outside, extracellular membrane). (**C**) Predicted hydrophobicity of the CcPI3KC3 amino acid sequence in common carp. (**D**) Prediction of the N-glycosylation of CcPI3KC3 in common carp. (**E**) Prediction of the O-glycosylation sites of CcPI3KC3 in common carp. (**F**) Prediction of the phosphorylation sites of CcPI3KC3 in common carp.

The prediction of the secondary structure was consistent with that of the tertiary structure (Figure 2). The CcPI3KC3 protein structure consisted of α-helix, β-sheet, β-turn, β-strand, and random coil structural motifs. Among them, α-helix and random coil were the main recognition motifs. However, the CcPI3KC3 protein structure had no signal peptides or transmembrane structures, and the hydrophobic value was −0.437, indicating that it is a hydrophilic, non-secretory protein (Figure 3A–C). As shown in Figure 3D, the amino acid sequence of CcPI3KC3 contained two potential N-glycosylation sites, but there were no O-glycosylation sites (Figure 3D,E). In addition, the prediction of the phosphorylation sites of the amino acid sequence showed that CcPI3KC3 contained 88 potential phosphorylation sites, with a threshold of 0.5, including 50 serine residues, 26 threonine residues, and 12 tyrosine residues (Figure 3F).

### 3.2. Phylogenetic Analysis

**Figure 4:**
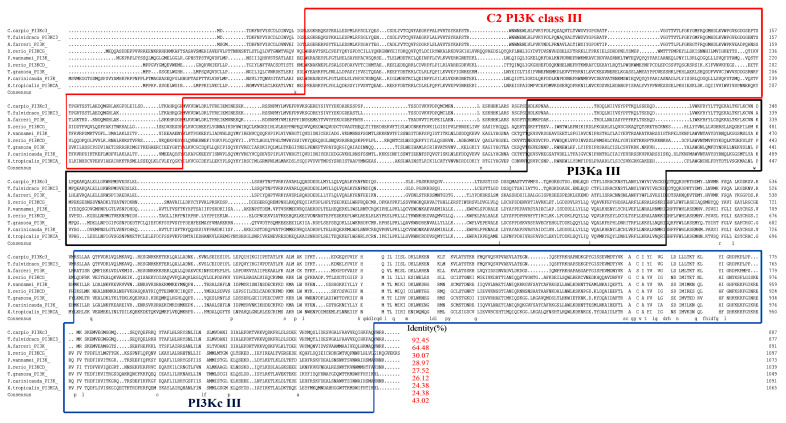
Multiple alignment of the deduced CcPI3KC3 amino acid sequence among common carp (*C. carpio*), yellow catfish (*T. fulvidraco*), scallop Chlamys farreri (*A. farreri*), zebrafish (*D. rerio*), blood clams (*T. granosa*), ridgetail white prawns (*P. carinicauda*), and tropical clawed frogs (*X. tropicalis*) was performed. The red frame represents the C2 PI3K class III domain, the black frame represents the PI3Ka III domain, and the blue frame represents the PI3Kc III domain. Purple represents 100% identity, red represents 75−100% identity, and blue represents 50−75% identity.

**Figure 5:**
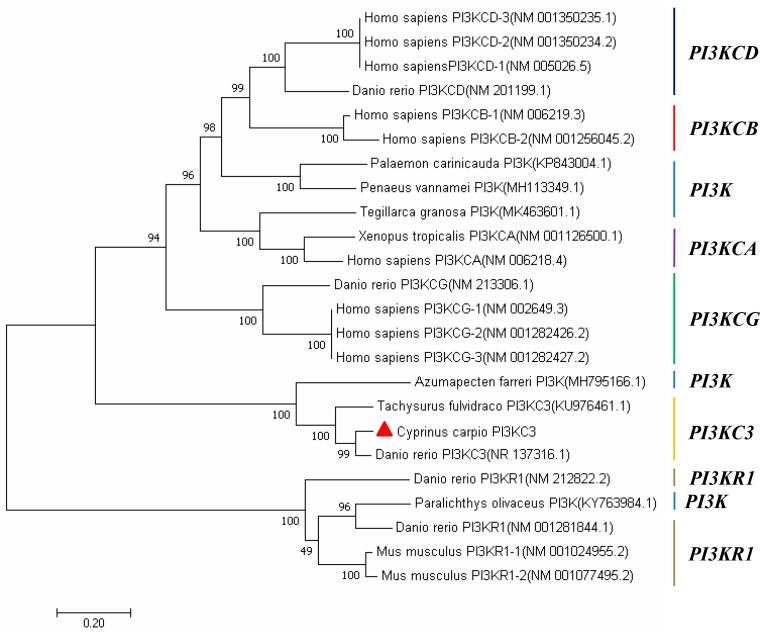
Phylogenetic analysis of the *CcPI3KC3* mRNA in common carp. The phylogenetic tree was constructed in the MEGA7 program via the neighbor—joining method with 1000 bootstrap replications. The GenBank accession numbers for the *PI3K* sequences are shown in brackets next to each species. Red triangle represents *CcPI3KC3 mRNA* in common carp.

The amino acid sequence of CcPI3KC3 was not similar to those of other bony fishes. The CcPI3KC3 was 92.45% similar to the orthologue encoded by yellow catfish and 64.48% similar to that encoded by zebrafish (Figure 4). Interestingly, the amino acid sequences of all the species included C2 PI3K class III (25–194 aa), PI3Ka III (291–503 aa), and PI3Kc III (539–889 aa) domains. Depending on the different catalytic subunits of PI3K, the phylogenetic tree was divided into three major clades (Figure 5). The mRNA sequence of *CcPI3KC3* clustered with those of yellow catfish and zebrafish, indicating that they were more closely related.

### 3.3. Expression of the CcPI3KC3 mRNA in Common Carp Tissues

**Figure 6:**
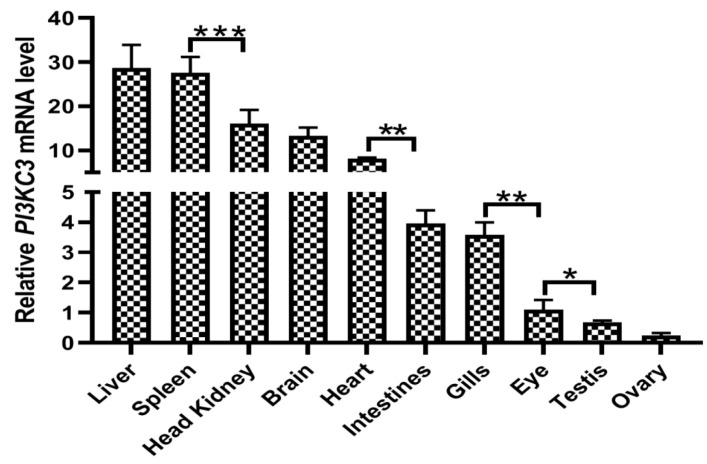
Relative expression levels of *CcPI3KC3* in different tissues (liver, spleen, head kidney, brain, heart, intestine, gills, eye, testis, and ovary) of German mirror carp (uninfected with CyHV-3). The mRNA expression level of the *CcPI3K* in the eye was considered to be 1. *β-actin* was used as an internal control. The data are shown as the means ± SDs of three technical replicates. The data were analyzed by one-way ANOVA (*n* = 3). Differences were considered statistically significant at *p* < 0.05 (* *p* < 0.05; ** *p* < 0.01, *** *p* < 0.001).

The results revealed that the relative expression of *CcPI3KC3* was different in liver, spleen, head kidney, brain, heart, intestine, gills, eye, testis, and ovary tissues (Figure 6). The highest level of *CcPI3KC3* mRNA expression was detected in the liver, followed by the spleen, head kidney, brain, heart, intestines, gills, eyes, testes and ovaries, in German mirror carp (uninfected with CyHV-3). Compared with those in other tissues, the relative expression levels of *CcPI3KC3* were significantly greater in the liver and spleen (*p* < 0.001) but significantly lower in the gonadal testes and ovaries (*p* < 0.05).

### 3.4. CcPI3KC3 mRNA Expression Patterns at Different Times after CyHV-3 Infection

**Figure 7:**
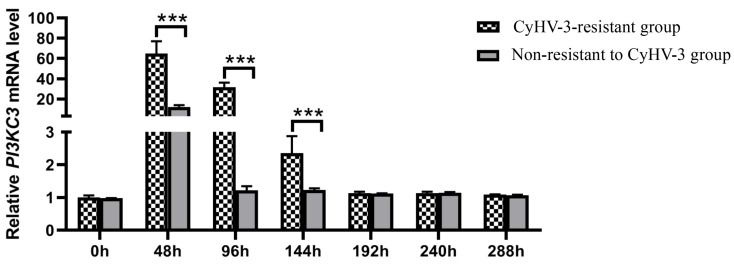
Expression analysis of *CcPI3KC3* after CyHV-3 infection (0, 48, 96, 144, 192, 240, and 288 hpi) in the head kidney tissues of the “Longke-11” mirror carp (CyHV-3-resistant group) and the German mirror carp (non-resistant to CyHV-3 group). The mRNA expression level of the *CcPI3K* in “Longke-11” mirror carp infected at 0 hpi was considered to be 1. The data represent the means ± SDs of three technical replicates. The data were analyzed by two-way ANOVA (*n* = 3), and statistically significant differences were defined at *p* < 0.05 (*** *p* < 0.001).

We determined the relative expression patterns of *CcPI3KC3* in “Longke-11” mirror carp and the German mirror carp at different times after CyHV-3 infection (Figure 7). With increasing CyHV-3 infection time, the expression of the *CcPI3KC3* mRNA showed a trend of first increasing and then decreasing. The relative expression level of *CcPI3KC3* in the “Longke-11” mirror carp group was significantly greater at 48, 96, and 144 hpi than that in the German mirror carp group after infection (*p* < 0.001). In addition, there was no significant difference in the relative expression levels of *CcPI3KC3* between the “Longke-11” mirror carp group and the German mirror carp group at 0, 192, 240, and 288 hpi.

## 4. Discussion

PI3K is a member of the lipid kinase family. At present, the signaling pathway related to PI3K is one of the most extensive activation pathways, and it has been proven that it can play an important role in cell proliferation, adherence, survival, autophagy, and motility [24,25]. The PI3K-Akt signaling pathway plays a vital role in regulating cell antiviral responses [26], and it is speculated that PI3K has inhibitory effects on viral replication during viral infection in the host. In this study, we determined for the first time the full-length sequence of *CcPI3KC3* in the PI3K family of common carp and revealed that it contains a 5′-UTR of 390 bp, a 3′-UTR of 265 bp, and an open reading frame of 2664 bp. Furthermore, the instability index of the CcPI3KC3 protein was 46.38, which was greater than 40. The protein functions are closely related to protein structure, and protein–protein interactions are related mainly to α-helix, β-corner, and β-folding structures, while random curly helps to identify post-translation correlation [27,28]. The predicted results of the secondary and tertiary structures of CcPI3KC3 were consistent and indicated that the protein contained mainly α-helices and random curls. Signaling peptides, which are mainly composed of hydrophobic amino acids, are mainly responsible for guiding the new synthetic proteins to the secretion channels and affecting protein transfer [29,30]. The CcPI3KC3 protein has no transmembrane region, consistent with previous results [31,32]. Studies have shown that N-glycosylation sites play important roles in protein immunity and antigens. N-glycosylation sites work to regulate the surface expression and function of proteins, affecting lymphocyte activation, apoptosis, antigen recognition, and clearance [33]. The CcPI3KC3 amino acid sequence contains two N-glycosylation sites, suggesting that the *CcPI3KC3* gene might play an important role in the immune response of lymphocytes in common carp. CcPI3KC3 was estimated to have 88 phosphorylation sites, mainly consisting of serine residues and threonine residues, which play important roles in posttranslational protein modification [34,35]. Thus, the prediction of protein functional regions and sites provides an important theoretical basis for subsequent functional studies of *CcPI3KC3* in common carp.

Protein domains are the basic units of protein folding, evolution, and function [36]. By studying similar domain compositions in different proteins, its functional characteristics can be inferred [37]. In this study, we found that CcPI3KC3 has a C2-binding (C2 PI3K class III) domain, a protein attachment (PI3Ka III) domain, and a c protein catalytic (PI3Kc III) domain. Among them, the C2 PI3K class III domain usually plays a role through Ca^2+^. It is a common typical domain in the protein that regulates signal transduction or membrane transport [38], whereas the PI3Kc III domain functions as an ATP binding site and catalytic effector [39]. The results of protein domain prediction in this study were similar to the results from the tongue sole (*Cynoglossus semilaevis*) [31,32,40]. The homology and phylogenetic tree results revealed that CCPI3KC3 and yellow catfish presented high homology and are clustered into one clade. Previous research has shown that PI3KC3 in yellow catfish is necessary for apoptosis and the key factors involved in the process of liver autophagy and inflammatory, which indicated that *CcPI3KC3* might also be involved in immune-related reactions in common carp [32,41]. Moreover, the phylogenetic tree of *PI3K* revealed that all the *PI3KC3* genes were clustered together, which indicated that *PI3KC3* is evolutionarily conserved and provides some supporting evidence for the evolutionary process of common carp.

Gene expression patterns in different tissues can be used as an important basis for gene function prediction. However, the *PI3KC3* expression profile in fish tissues has rarely been studied. In the common carp, *CcPI3KC3* was widely distributed across ten tissues with different expression levels. Additionally, the mRNA expression levels of *CcPI3KC3* in the liver, spleen, and head kidney tissues were significantly greater than those in other tissues (*p* < 0.001), suggesting that *CcPI3KC3* might play key roles in the immunological processes of the liver, spleen, and head kidney in common carp. Similarly, some studies on other fish have shown that the mRNA of *PI3K* family genes are highly expressed in liver, spleen and kidney tissues [32,38]. The liver, spleen, and kidneys are important immune organs of fish and are closely related to fish health [42,43,44]. Therefore, our study suggested that *CcPI3KC3* plays an important role in the activation of lymphocytes and the secretion of immune factors in the immune organs of common carp.

After common carp are infected with CyHV-3, they will first experience a series of immune responses. The virus is able to induce autophagy in cells to promote viral replication. Autophagy blocks retinoic acid-inducible gene protein (IRIG-1) mediated antiviral signaling and inhibits type I interferon (IFN) promoter activity to promote CyHV-3 replication [45]. On the other hand, CyHV-3 can promote autophagy in cells at an early stage, but then inhibit autophagy through mTOR at a later stage during CyHV-3 infection [46,47]. Recent studies have shown that phosphorylation of PI3K is necessary for the early initiation of autophagy when stimulated by external factors [48,49,50,51]. Research has also demonstrated that the PI3K-AKT signaling pathway can regulate autophagy levels, thereby inhibiting viral replication during the viral infection stage [15,38,52]. In our previous research, “Longke-11” mirror carp, a new mirror carp variety resistant to CyHV-3 infection, was determined to have a relatively high survival rate [18,53]. With the increase of infection time, the expression of *CcPI3KC3* mRNA initially increased but then decreased to a stable level, reflecting the trend of the relatively expression levels of CyHV-3 genes reported in previous studies [53]. More importantly, in the early stages of CyHV-3 infection, the expression of the *CcPI3KC3* gene in the head kidney of “Longke-11” mirror carp was significantly greater than that in the head kidney of German mirror carp during the early stages of CyHV-3 infection. Therefore, we further assume that *CcPI3KC3* participates in the early antiviral immune response to CyHV-3 infection, and might inhibit viral replication by reducing host’s autophagy. Overall, our study provides a new perspective for screening anti-CyHV-3 functional genes in common carp.

## 5. Conclusions

In conclusion, the full-length sequence of *CcPI3KC3* from common carp was cloned for the first time and molecularly characterized. The tissue expression profile revealed that *CcPI3KC3* mRNA was highly expressed in the liver, spleen, and head kidney tissues of common carp. In addition, the relative expression levels of *CcPI3KC3* differed at various CyHV-3 infection times and were significantly greater in “Longke-11” mirror carp (CyHV-3-resistant carp) and German mirror carp (non-resistant to CyHV-3) at the early stages of CyHV-3 infection. Thus, we concluded that *CcPI3KC3* may be involved in anti-CyHV-3 infection in common carp during the early stage of viral infection and that the mechanism of this effect may be related to autophagy.

## Figures and Tables

**Table 1:** Sequence of the primers used in the study.

Primers	Primer Sequences (5′-3′)	Application
*CcPI3KC3–1*	F:ATTCAAGTCACTGCGGTTATCGA	Conserved sequence amplification
	R:TGCATTAGGTTTGAGATCGTGGT	
*CcPI3KC3–2*	F:CCCTCGTGTCAAAAACGGTG	Conserved sequence amplification
	R:ATGTTTGAAGATGACAGGATATTGC	
*CcPI3KC3–3*	F:CCTGCTGGCTGATAATGAGAAG	Conserved sequence amplification
	R:GTTGGAATCTTTATTTGGCTGTCTT	
5′adaptor	F:GCTGTCAACGATACGCTACGTAACG	Nested 5′-race PCR
	R:GCATGACAGTGGGIIGGGIIGGGIIG	
3′adaptor	F:GCTGTCAACGATACGCTACGTAACGGCA	Nested 3′-racePCR
	R:TGACAGTGTTTTTTTTTTTTTTTTTT	
5.3′outer	GCTGTCAACGATACGCTACGTAAC	Nested PCR
5.3′inner	GCTACGTAACGGCATGACAGTG	Nested PCR
RC947-F3	AGGAGGCGGTGCATTATATGC	Amplification of the partial cDNA and nested 3′-race PCR
RC947-F4	ATTGATGAGAGTGTAGGTGCTCTGT	Amplification of the partial cDNA and nested 5′-race PCR
RC947-RT1	GACGTACAGGTCGGAGCAGC	5′RACE Amplification of the partial cDNA
RC947-RT2	GAGTGCTGAAGGCCTTGTAAGAC	5′RACE Amplification of the partial cDNA
RC947-R3	TTCTTGATACGACTCTGTCTGATTAATG	5′RACE Amplification of the partial cDNA
RC947-R4	GCTGATCAAAAGTACTGTCAGTCTTAAT	5′RACE Amplification of the partial cDNA
*qPI3KC3*	F:GGATTCCTTGGAGCTGCTGT	RT–PCR
	R:CACCAGCTGGAGCAAGTACA	
*β-actin*	F:GCCGTGACCTGACTGACTACCT	RT–PCR
	R:GCCACATAGCAGAGCTTCTCCTTG	

## Data Availability

Data will be made available upon request.
